# Biotherapies in Uveitis

**DOI:** 10.3390/jcm9113599

**Published:** 2020-11-08

**Authors:** Mathilde Leclercq, Anne-Claire Desbois, Fanny Domont, Georgina Maalouf, Sara Touhami, Patrice Cacoub, Bahram Bodaghi, David Saadoun

**Affiliations:** 1 Department of Internal Medicine, Hôpital Charles Nicolle, F-76000 Rouen, France; mat3leclercq@gmail.com; 2 Department of Internal Medicine and Clinical Immunology, AP-HP, Centre national de références Maladies Autoimmunes et systémiques rares et Maladies Autoinflammatoires rares, Groupe Hospitalier Pitié-Salpêtrière, F-75013 Paris, France; anneclaire.desbois@aphp.fr (A.-C.D.); fanny.domont@aphp.fr (F.D.); georgina.maalouf@aphp.fr (G.M.); patrice.cacoub@aphp.fr (P.C.); 3 Sorbonne Universités, UPMC Univ Paris 06, INSERM, UMR S 959, Immunology-Immunopathology-Immunotherapy (I3), F-75005 Paris, France; 4 Biotherapy (CIC-BTi), Hôpital Pitié-Salpêtrière, AP-HP, F-75651 Paris, France; 5 Department of Ophthalmology, AP-HP, Groupe Hospitalier Pitié-Salpêtrière, F-75013 Paris, France; sara.touhami@aphp.fr (S.T.); bahram.bodaghi@aphp.fr (B.B.)

**Keywords:** non-infectious uveitis, biotherapy, anti-TNF-α (anti-tumor necrosis factor alpha) agent, tocilizumab, Janus Associated Kinase (JAK) inhibitors

## Abstract

Non-infectious uveitis (NIU) represents one of the leading causes of blindness in developed countries. The therapeutic strategy aims to rapidly control intra-ocular inflammation, prevent irremediable ocular damage, allow corticosteroid sparing and save the vision, and has evolved over the last few years. Anterior NIU is mostly managed with topical treatment in adults. However, for intermediate, posterior and pan-uveitis, notably when both eyes are involved, systemic treatment is usually warranted. Biotherapies are recommended in case of inefficacy or non-tolerance of conventional immunosuppressive drugs in non-anterior NIU. Anti-tumor necrosis factor alpha (anti-TNF-α) agents are by far the most widely used, especially adalimumab (ADA) and infliximab (IFX). In case of sight-threatening uveitis in Behçet’s disease or in case of risk of severe recurrences, respectively IFX and ADA may be recommended as first-line therapy. Many questions are left unanswered; how long to treat NIU, how to discontinue anti-TNF-α agents, what biologic to use in case of anti-TNF-α failure? The objective of this review is to present an updated overview of knowledge on the use of biological treatments in NIU.

## 1. Introduction

Uveitis is an heterogenous nosological entity. The uvea corresponds to the intermediate membrane of the ocular bulb, including the choroid (nutritious vascular tissue), the ciliary bodies and the iris [1]. However, the term uveitis is broader, encompassing inflammatory damage to the retina and its vessels and the papilla. Uveitis is classified anatomically as anterior uveitis (anterior ciliary bodies and iris), intermediate (posterior ciliary bodies, vitreous humor and pars planitis) or anterior and intermediate uveitis and posterior (retina and choroid). Pan-uveitis involves the three compartments, without predominant site. Although, uveitis classification distinguishes infectious disease from non-infectious uveitis (NIU) [2]. In tertiary center, one third of patients have non-infectious inflammatory uveitis [3]. This inflammation can be systemic (sarcoidosis, Behçet’s disease) or limited to the eye (birdshot chorioretinopathy) [4]. NIU is the main cause of uveitis in industrialized countries, with a prevalence of 121/100,000 persons [5]. Uveitis appears as the fifth most common cause of visual loss in developed countries, affecting young people (60–80% of patients are between 20 and 50 years old) [6]. This poor visual prognosis is secondary to the development of ocular complications. Patients with uveitis will more frequently develop cataracts (25% higher risk) or glaucoma (15% higher risk) than general population [7]. Thus, management of NIU and its complications is a new challenge.

Therapeutic strategy has evolved over the last few years. Anterior NIU is mostly managed with topical treatment in adults. However, for intermediate, posterior and pan-uveitis, notably when both eyes are involved, systemic treatment is warranted. Corticosteroids (intravenous methylprednisolone and/or oral prednisone) represent the first-line of treatment [2]. Conventional immunosuppressive drugs are recommended in cases of persistent or severe inflammation to limit the occurrence of complications, in cases of relapse during corticosteroid tapering or in cases of corticosteroid dependency [8]. The Systemic immunosuppressive therapy for eye diseases (SITE) cohort studies [9] have shown the efficacy of methotrexate [10], mycophenolate mofetil [11] and azathioprine [12] in resolution of intra-ocular inflammation and corticosteroid sparing effect. In a retrospective study, Gangapura et al. compared efficacy of methotrexate (median dose of 12.5 mg/week) versus mycophenolate mofetil (median dose of 1 g twice daily) in 352 patients [13]. The two groups were not comparable for uveitis localization and presence of macular edema. The authors showed that mycophenolate mofetil improved ocular inflammation more quickly and had higher corticosteroid sparing effect. However, the failure rate under immunosuppressive drugs remains around 30% [13]. In front of this reality and thanks to a better knowledge of the mechanisms involved in intra-ocular inflammation, biotherapies have emerged. Several randomized prospective studies have enabled some of these molecules to obtain Food and Drug Administration (FDA) approval for the treatment of uveitis.

The aim of this review is to provide an updated understanding of the use of biological therapies in non-infectious uveitis.

## 2. Pathophysiological Rationale

Uveitis mice models, such as experimental autoimmune uveitis (EAU), allowed better understanding of uveitis inflammatory origin. CD4+ T-cells are the main lymphocytes cells found in humor aqueous samples [14]. Both Th1 and Th17 T-cells are involved in uveitis mechanisms. It has been observed that the transfer of Th1 and Th17 T-cells induces uveitis in wild mice [15]. Th1 T-cells are mostly involved in intra-cellular bacteria destruction or viral response and secreted interferon gamma (IFNγ). Th17 T-cells are mostly involved in extra-cellular bacteria and fungus reactions. A high level of tumor necrosis factor alpha (TNF-α) was found in humor aqueous of uveitis mice models. This cytokine is responsible for ocular infiltration by T lymphocytes and macrophages. Blockage of TNF-α and its receptor is effective in the control of intra-ocular inflammation in mice models of EAU [15]. Knock-out mice for TNF-α receptor showed a decrease in the number of immune cells in EAU models [16].

IL-6 is an important cytokine in uveitis. Murine models deficient in IL-6 will develop a less severe disease [17]. IL-6 also plays a role in vascular exudation phenomena by promoting abnormal secretion of vascular endothelial growth factor (VEGF), implied in the formation of macular edema [18]. Finally, in EAU models, a defect in the production of regulatory cytokines, such as IL-10, has been reported [17]. In humans, elevated levels of TNF-α, IL-17, IL-1 and IL-6 have been found in the aqueous humor of patients with birdshot chorioretinopathy, Vogt-Koyanagi-Harada disease and Behçet’s disease [17]. New studies have focused on a possible trigger of ocular inflammation from the gut bacterial microbiome [19].

## 3. Anti-TNF-α Agents

The main literature results on anti-TNF-α agents are reported in Table 1.

### 3.1. Adalimumab

Adalimumab (a fully humanized monoclonal antibody that blocks the interaction between TNF-α and TNF R1 and TNF R2 receptor) and infliximab (a chimeric monoclonal antibody directed against TNF-α) were mostly studied in uveitis treatment [20].

The efficacy of adalimumab (ADA) in rapid control of inflammation [21], prevention of relapse and corticosteroid sparing [22] has been shown in two prospective randomized studies. In the VISUAL I study, 217 patients with active uveitis (despite the use of prednisone 10 to 60 mg for 2 or more weeks before) were randomized: 110 patients received ADA and 107 controlled patients had placebo. Corticosteroid was tapered over 16 weeks. The median time to observe treatment failure was 24 weeks in the ADA group and 13 weeks in the placebo group. ADA avoided 50% of treatment failures (hazard ratio, 0.50; 95% confidence interval 0.36 à 0.70; *p* < 0.001). Patients treated with ADA had less anterior and vitreous inflammation under treatment [21]. In the VISUAL II study, 229 patients with cortico-dependent inactive uveitis were randomized: 115 patients received ADA and 114 controlled patients had placebo. Corticosteroid was tapered off over 19 weeks. The median time to observe treatment failure was > 18 months in the ADA group and 8.3 months in the placebo group. Rates of treatment failure because of anterior or vitreous inflammation were not different between the two groups [22]. The results of the VISUAL studies show the effectiveness of ADA in the control of ocular inflammation, but a few points for reflection and discussion can be highlighted. The prednisone tapering schedule within 16 and 19 weeks respectively used in these two studies is questionable since, in many countries, prednisone is usually maintained for several months before withdrawal, as recommended by previous guidelines and experts. In VISUAL I study, the efficacy of ADA was not greater than that of placebo in the subgroup of patients who were using immunosuppressive agents. In the VISUAL III study [23], a prospective phase IV study including 371 patients from the VISUAL I and VISUAL II studies, 242 (65%) patients had active uveitis. At the end of the study (78 weeks), 40% were still active.

In a large prospective study of 131 patients, Díaz-Llopis et al. [24] showed the efficacy of ADA in controlling anterior and posterior inflammation after 6 months of treatment. All patients had refractory uveitis to at least one immunosuppressive drug. Patients had pan-uveitis (43.5%), juvenile idiopathic arthritis (JIA) (29.7%) or idiopathic uveitis (20.6%). During the 6 months of follow-up, 38.2% of the patients experienced a relapse. A complete resolution of macular edema occurred in 70% of the patients. Eighty-five percent of the patients could reduce 50% of their baseline immunosuppressive drugs.

Vallet et al. [25] retrospectively studied the efficacy of ADA and infliximab (IFX) in 160 refractory uveitis (pan-uveitis in 62%). Behçet’s disease (36%) was the main cause. Most of the patients had concomitant treatment with corticosteroid (84%) and conventional immunosuppressive drugs (64%). The overall rate of efficacy was 87% at 6 months, 93% at 12 months and 95% at 24 months. However, the incidence of complete response was 26% at 6 months, 28% at 12 months and 29% at 24 months. Event-free survival was 90% at 6 months, 70% at 12 months and 59% at 24 months. A significative decrease on corticosteroid dose was observed. Using a propensity score analysis, no significant difference was noted between ADA and IFX in terms of safety and efficacy. Factors associated with complete response to anti-TNF-α were the occurrence of more than five relapses before initiation of anti-TNF-α treatment and Behçet’s disease [25].

In a recent open-label multicenter study, Atienza-Mateo et al. [26] studied the efficacy of IFX and ADA in refractory Behçet’s uveitis. Seventy-four patients were treated with ADA and 103 patients received IFX. Improvement of anterior chamber inflammation, vitritis, retinal vasculitis, macular thickness and visual acuity after one year of treatment was highlighted in both groups.

The efficacy of ADA has been shown in juvenile idiopathic arthritis (JIA) in two prospective studies. In SYCAMORE, a randomized controlled study [27], patients with persistent ocular inflammation despite methotrexate and corticosteroid were included: 60 patients were treated with ADA and 30 patients received placebo. Treatment failure rate was significantly lower with ADA compared to placebo (27% versus 60%, *p* = 0.002). Moreover, patients treated with ADA had a significant reduction in corticoids drops. The ADJUVITE study confirmed these results on 31 patients, with a rapid control of ocular inflammation after two months of treatment [28].

### 3.2. Infliximab

IFX is a chimeric monoclonal antibody directly against TNF-α. IFX has shown its efficacy in the management of NIU through prospective open-label studies. In a prospective study on 32 patients, mostly with idiopathic uveitis, Suhler et al. [29] found a sustained efficacy of IFX: 77% of efficacy after 10 weeks of treatment and 60% after two years of treatment. In a study of 10 patients with active Behçet’s disease, the authors showed early and rapid efficacy of IFX in reducing intraocular inflammation and improving visual acuity [30]. In a retrospective study of 23 patients with intermediate idiopathic uveitis, 82.6% of patients achieved remission after three months of treatment [31]. IFX appears to be effective in the resolution of macular edema (100% of 25 patients) and retinal vasculitis (94.9% of 39 patients) in Behçet’s disease [32]. IFX can control severe refractory uveitis: 30.8% of uveitis that failed to respond to azathioprine, cyclosporine and prednisone remained attack-free with IFX, with significantly lower uveitis attacks [33]. For long term retention rate, Fabiani et al. found that IFX retention rate in Behçet’s uveitis was 86.2% at 24 months, 75.7% at 60 months and 47.1% at 120 months [34]. After 10 years, 15 patients (37.5%) had discontinued IFX: 53% because of treatment failure and 13% because of disease remission. In a retrospective study of 164 patients, Takeuchi et al. [35] observed an improvement of visual acuity in 55% of the patients and a significant decrease of uveitis relapse during IFX treatment. Ohno et al. [36] found that efficacy of IFX was significantly lower in patients with longer disease duration, those with comorbid diabetes mellitus and those with less severe uveitis.

Multiple international experts recommended IFX as first-line therapy in the management of sight-threatening Behçet’s disease [2,37,38].

### 3.3. Other Anti-TNF-α Agents

Golimumab is a fully humanized monoclonal antibody [39]. Golimumab appeared to be effective in controlling ocular inflammation and improving visual acuity after 6 months [40] or 2 years of treatment [41]. In a multicentric study, complete remission was observed in 87% of the 15 patients treated with golimumab [41]. Interestingly, Fabiani et al. [42] showed, in Behcet’s uveitis, a complete control of intraocular inflammation in 87.5% of the patients after 12 months of follow-up. Moreover, resolution of vasculitis was observed in all patients after 3 months of treatment. Golimumab also controlled JIA uveitis with both efficacy in improvement of visual acuity, control of ocular inflammation, reduction of macular edema and corticosteroid sparing effect [43].

Certolizumab is a recombinant humanized monoclonal antibody [39]. Rudwaleit et al. showed a decrease in uveitis flares with certolizumab, compared to placebo [44]. Certolizumab efficacy ranged from 60% [45] to 71.4% [46]. In the study of Llorenç et al. [46], 42.8% of patients with chronic uveitis could withdraw from corticosteroid. In this study, 28.5% of the patients showed an improvement of visual acuity and for 57.1%, visual acuity remains stable. Tosi et al. [47] retrospectively studied 10 patients treated with golimumab and 11 with certolizumab for refractory uveitis. The number of ocular flares decreased from 128.6 events for 100 patients-year to 42.9 events for 100 patients-year during the first year of treatment.

Etanercept is a fusion protein of the extracellular domains of p75 and p55 TNF-α receptor and the Fc fragment of human immunoglobulin G. International guidelines have concluded that there is no evidence to support the use of etanercept in the management of uveitis [2,8,48]. In a prospective study including 20 patients controlled with methotrexate, etanercept was not efficient in preventing relapses and improving visual acuity during methotrexate tapering [49]. Flare-up of uveitis has been reported under etanercept. Meta-analyses have shown the inferiority of etanercept over other anti-TNF-α agents in uveitis [50].

### 3.4. Recommendations and Outstanding Questions

#### 3.4.1. What Are the Recommendations for the Use of Anti-TNF-α Agents in Uveitis?

In adults, ADA is now approved by the FDA and the European Medicine Agency (EMA) for the treatment of patients suffering from non-infectious non-anterior uveitis (NINAU) in case of cortico-dependence or contraindication to corticosteroid. International experts recommend the use of ADA in case of inefficacy or non-tolerance of conventional immunosuppressive drugs in NINAU [2,8]. IFX is recommended as first line therapy in case of sight-threating uveitis in Behçet’s disease (severe vasculitis, macular ischemia, cystoid macular edema, monophthalmic patient) [48]. In case of axial symptomatic spondylo-arthritis, anti-TNF-α agents are recommended in case of recurrent anterior uveitis (>3 relapses/year) [37].

In children, ADA is approved by the FDA in the treatment of chronic non-infectious anterior uveitis from the age of two years and adolescents in case of insufficient response or intolerance to conventional treatment, such as methotrexate, or for whom conventional treatment is inappropriate [51]. In JIA, anti-TNF-α agents are also recommended in case of topical corticosteroid dependency despite methotrexate [48].

ADA and IFX appear to be well tolerated. In a meta-analysis, Ming et al. reported that 30.6% of the patients had adverse events [52], with an incidence of 9.6 events/patient/year. Most frequent adverse events were reaction to infusion (3–17%), infectious diseases (6%), including tuberculosis, and occurrence of demyelinating or autoimmune diseases was seldom reported [53].

We propose to summarize the different actual recommendations for the use of anti-TNF-α agents of NIU (Table 2).

#### 3.4.2. What Is the Long-Term Efficacy of Anti-TNF-α Agents?

Llorenç et al. [54] analyzed 392 patients treated with ADA, mostly for non-anterior uveitis (62%). The drug retention rate was 92.97% at 6 months, 87.68% at 12 months, 76.31% at 24 months and 54.28% at 60 months. The same results were observed in Bitossi’s study [55]. In Llorenç’s study [54], 151 (38.5%) patients discontinued ADA during the follow-up (median time 49 months): 18.6% following a lack of efficacy and 8.7% due to side effects. Median drug retention time was 18.7 months in patients with inefficacy. Patients treated with >7.5 mg/day of prednisone and receiving ADA as a secondary biotherapy had a significantly shorter drug retention time. The drug retention rate was not different in patients treated with ADA as monotherapy or with concomitant immunosuppressive drugs. Interestingly, only 6.4% of the patients discontinued the treatment for sustained quiescence [54]. Fabiani et al. compared ADA and IFX retention rates and found no significant difference [56]. Vallet et al. found no significant difference between ADA and IFX in terms of safety and efficacy [25].

At 6 months, the relapse rate varied from 7% [25] to 38% [24]. Sharma et al. [57] studied 9 patients treated with ADA and 34 with IFX, and found that 67% of ADA patients had a relapse, with a median time of 2 years, whereas 53% of IFX patients had a relapse, with a median time of 3.4 years. In Behçet’s uveitis, Takeuchi et al. [35] highlighted uveitis relapses in 59.1% of patients during IFX treatment, with a mean time to relapse of 8.5 months. Eighty percent of the relapse occurred during the first year of treatment. Al-Janabi et al. [58] studied long-term efficacy of ADA (60 patients) and IFX (76 patients). The main etiology was Behçet’s disease. They showed that disease flare (defined as intraocular inflammation at least six months after previous controlled inflammation needed new treatment) occurred in 42.3% of eyes, with a median time to first flare of 5.4 years. Treatment failure (repeat flares ≥2 or serious side effects needing treatment discontinuation) occurred in 24.3% with a 5-years survival rate of 68%.

Thus, ADA failure rate remains high, at around 30%. The majority of treatment discontinuation is related to treatment failure, while the percentage of withdrawal for sustained efficacy remains low. The risk factors identified are, on the one hand, the severity of the uveitis and, on the other hand, the use of ADA as a secondary biotherapy. Most treatment failure occurs in the first months of treatment, before one year of treatment.

#### 3.4.3. How to Manage the Failure of an Anti-TNF-α Agent

One of the options is to switch to another anti-TNF-α agent. The efficacy of ADA after IFX inefficacy has already been reported [24,59]. In Vallet’s study of 124 Behçet’s patients, 37 patients received second line anti-TNF-α agents secondary to lack of efficacy or side effects. After anti-TNF-α agents switch, complete response at 6 months was observed in 67% of the patients [32]. The same percentage was observed in Olivieri’s study [60]. In JIA, Simonini et al. [61] found that the switch from IFX to ADA, secondary to treatment failure, allowed 69.6% of disease control after 6 months of treatment.

Another option in case of inefficacy is dose escalation. In Sukumaran’s study, 35% of patients need a dose escalation ≥ 10 mg/kg of IFX, according to disease control for 80% of patients [62]. In case of inefficacy, Takeuchi et al. proposed shortening the interval of IFX infusion in 22% of cases or to increase IFX dose in 2% of cases [35]. Two recent studies showed that 56% [63] and 67% [64] of refractory patients, respectively, were controlled after weekly ADA administration.

The last option is to switch to another biotherapy. Tocilizumab, an anti-IL6 receptor, has shown the most promise results in the strategy. Disease control was observed in 60–70% of refractive patients, including JIA, six months after introduction of tocilizumab [65,66,67].

#### 3.4.4. Is It Interesting to Add Conventional Immunosuppressive Drugs to Anti-TNF-α Agents?

Several studies have focused on the risk of developing anti-drugs antibodies and on the interest in concomitant treatment with conventional immunosuppressive drugs, such as methotrexate or mycophenolate mofetil, to prevent their development. In a multicentric study of 595 patients with rheumatoid arthritis, 31.2% of patients treated with ADA and 17.4% treated with IFX developed anti-drugs antibodies [68]. Patients with anti-drugs antibodies had a decreased level of drug exposure and were less often in remission. In uveitis, permanent anti-ADA antibodies were detected earlier after starting with ADA and were associated with a low serum level of ADA and a worse uveitis outcome [69]. In JIA, Marino et al. [70] showed that 37% of patients had anti-ADA antibodies. These patients had significantly more relapses than patients without antibodies. However, these results remain to be discussed [71]. Some authors in chronic inflammatory diseases suggested that the adjunction of immunosuppressive drugs decreased anti-drugs antibodies and increased serum drugs levels [72]. However, it is unclear whether concomitant non corticosteroid immunosuppressive drugs limit the development of these antibodies and promote a better therapeutic response [69]. In VISUAL I [21], patients treated with ADA and conventional immunosuppressive drugs had a relapse rate and time to treatment failure comparable to patients treated with ADA without those treatments.

#### 3.4.5. What Is the Duration of Therapy with Anti-TNF-α Agents?

Very few studies have focused on this point. Actually, there is no consensus statement on treatment duration in NIU [8], except for severe Behçet’s uveitis where experts recommended a minimum of two years of remission before considering reducing treatment [37]. In Llorenç’s study, most patients stopped the treatment due to efficacy at 60 months. After stopping treatment for efficacy, relapse risk remains high. Shakoor et al. [73] showed a relapse rate of 61.1% after stopping IFX, with a median time to relapse of 20 months. In Behçet’s disease, 40–50% of patients had maintained remission after IFX discontinuation with a median follow-up from 7.5 months to 3 years [74,75]. French experts recommended at least one year of treatment, with the exception of Behçet’s uveitis where longer duration is mandatory [48].

#### 3.4.6. How to Discontinue an Anti-TNF-α Agent

Interestingly, Martín-Varillas et al. [76] proposed a therapeutic strategy to discontinue ADA in Behçet’s uveitis. After one year of ADA therapy and three to six months of disease remission, the authors proposed increasing the spacing between each injection by one week. If effective for three months, the authors proposed continuing the spacing by another week, up to one injection every 6 weeks. Then the treatment was stopped. In the non-optimized group, treatment was maintained with the same periodicity of injection (ADA every 2 weeks). This strategy allowed a control of ocular inflammation, and an improvement of visual acuity, vasculitis and macular edema, similar to the results obtained in the non-optimized group. Moreover, the optimized group observed less adverse events and had a lower mean treatment cost [76]. Several studies have shown the efficacy of spacing IFX or ADA injections in rheumatoid arthritis, with a sustained remission of 40 to 60% during withdrawal [77]. International experts recommend the spacing of anti-TNF-α agents in rheumatoid arthritis [78] and psoriatic arthritis [79]. This interesting strategy should be considered for NIU.

#### 3.4.7. Which Anti-TNF-α Agent, ADA or IFX, Is Recommended in Uveitis?

ADA and IFX are the main anti-TNF-α agents used in uveitis. Some uncontrolled studies have compared ADA and IFX efficacy. In an open-label study, Atienza-Mateo et al. [26] compared the efficacy of ADA and IFX in 177 Behçet’s uveitis cases. During the year of follow-up, the authors showed no difference between the two groups in terms of efficacy, relapse rates or serious side effects. Visual acuity was significantly higher in the ADA group after one year of treatment, but the authors did not use the LogMAR unit to compare visual acuity [26]. In a multicenter retrospective study of 160 patients, Vallet et al. [25] found a trend towards superiority of ADA in terms of event-free survival (*p* = 0.08), with a trend towards more adverse events with IFX. On the other hand, Fabiani et al. [80] observed a trend towards superiority of IFX in corticosteroid sparing effect and reduction of macular edema. In childhood uveitis, ADA seems to be more efficient for sustained disease control [81]. Further prospective randomized studies will be needed to reach definitive conclusions.

In a recent post-hoc analysis of VISUAL I and II studies [82], the authors showed that idiopathic uveitis treated with ADA had a lower risk of treatment failure compared to patients receiving placebo. However, the risk was not statistically different for other etiologies like birdshot chorioretinopathy or Behçet’s disease.

#### 3.4.8. What Is the Efficacy of Anti-TNF-α Agents in Sight Threatening Uveitis?

The efficacy of anti-TNF-α agents on retinal vasculitis has emerged from Behçet’s disease studies. Calvo-Río et al. have shown a decrease of 91% of retinal vasculitis during one year of follow-up in Behçet’s disease [83]. A significant, rapid and prolonged reduction in vasculitis lesions has been reported several times with IFX [84] or ADA [85] in Behçet’s disease. Vallet et al. found an improvement of 93.9% of vasculitis lesions with anti-TNF-α agents. However, retinal vasculitis was negatively associated with complete response to anti-TNF-α treatment in multivariate analysis [32].

A recent editorial highlighted the challenge of evaluating macular edema as trial endpoint [86], because it is a major cause of visual loss function during uveitis [58]. In a retrospective study of 25 patients [87], resolution of macular edema was observed in 50% of patients with ADA and 56% of patients with IFX after 24 months of treatment. Díaz-Llopis et al. [24] found complete resolution of macular edema in 70% of cases after 6 months of treatment with ADA. In contrast, in the VISUAL III study [23], stability of central macular thickness was noted during follow-up. Interestingly, a Cochrane review [88] reported that no prospective study has focused on resolution of macular edema with anti-TNF-α agents. In the VISUAL I [21] and VISUAL II [22] studies, the risk of developing macular edema on ADA was significantly decreased compared to the placebo group, but the difference between the two groups in the time to evidence of macular edema was not significant. However, the course of macular edema under treatment was not reported at all. Further prospective studies are needed.

#### 3.4.9. How to Manage Corticosteroids and Conventional Immunosuppressive Drugs in the Era of Anti-TNF-α Agents?

The MUST (Multicenter uveitis steroid treatment) [89] study showed, after seven years of follow-up, a superiority of conventional immunosuppressive treatments or biotherapies over a uni- or bilateral fluocinolone implant. This superiority concerned the improvement of visual acuity. In the group of patients treated with implants, there was an 8% increase in the number of patients with blindness (visual acuity ≤ 1/10) at 7 years compared to treatment initiation. In the systemic therapy group, a 1% decrease in the number of blind patients was shown. The beneficial effect of immunosuppressive treatment also included improvement in initial cystoid macular edema and ocular side effects (cataract and glaucoma) [89]. An economic evaluation of ADA and dexamethasone intravitreal implant (OZURDEX) showed an incremental cost-effective ratio (ICER) of £19,509 per quality-adjusted life-year (QALY) gained for OZURDEX. The ICER of ADA was £94,523 per QALY gained in active uveitis and £317,547 in inactive uveitis per QALY gained. These results should be discussed along with the long-term efficacy of ADA compared to dexamethasone intravitreal implant [90]. In a retrospective study, comparison of efficacy between conventional immunosuppressive drugs and anti-TNF-α agents showed no significant difference between the two treatments in terms of treatment failure, corticosteroid sparing effect, visual acuity improvement or adverse events [91]. However, anti-TNF-α agents allowed a quicker control of ocular inflammation.

## 4. Anti-IL6 Agents

Tocilizumab (TCZ) is a humanized monoclonal antibody which inhibits IL-6 signaling by preventing IL-6 from binding to its receptor. TCZ is approved in the treatment of rheumatoid arthritis, Still’s disease and giant cells arteritis [92]. Two prospective trials studied the efficacy of TCZ in non-infectious uveitis. STOP-uveitis included 37 non-anterior uveitis: 18 patients received 4 mg/kg TCZ and 19 patients received 8 mg/kg [93]. Most of the uveitis (76%) were idiopathic and 59.4% of the patients received TCZ as first line therapy (naive patients), without exposition to corticosteroids. Active uveitis was only definite on vitreous haze. There was no standardization in corticosteroid decrease and only 18.9% of the patients had corticosteroids at baseline. The authors observed a significant improvement in visual acuity and a reduction of central foveolar thickness. A two-step decrease in vitreous haze was experienced by 43% of patients. There was no significant difference between the two doses of TCZ, neither between naive patients nor other patients. TCZ, administrated subcutaneously, has also been studied in JIA in the APTITUDE study [94]. This was a multicenter single-arm study including 21 patients with active uveitis, refractory to anti-TNF-α agents. Patients treated with corticosteroids > 0.2 mg/kg were excluded. Primary efficacy end point was efficacy, definite using SUN criteria, after 12 weeks of treatment. At 12 weeks, 33% of patients achieved treatment response and 29% discontinued the treatment before the first 3 months, mostly because of inefficacy. The study did not meet the prespecified criterion at 12 weeks to justify a phase 3 trial. However, the efficacy of TCZ at 8 mg/kg has been shown in a retrospective study of 25 patients with refractory uveitis in JIA [67]. There was a significant increase of visual acuity and decrease of intra-ocular inflammation after 6 and 12 months of treatment. Interestingly, TCZ improved retinal vasculitis lesions and all the patients with cystoid macular edema had a normalization of macular thickness after 6 and 12 months of treatment.

TCZ has also shown its efficacy in refractory Behçet’s uveitis [95]. Atienza-Mateo et al. [96] showed a rapid improvement of ocular inflammation and visual acuity in 11 refractory uveitis patients to at least one anti-TNF-α agent. Interestingly, there was a resolution of all retinal vasculitis during follow-up. TCZ was also effective in controlling macular edema in birdshot chorioretinopathy [97].

TCZ seems to be particularly effective in reduction of macular edema. In a retrospective study on macular edema refractory to conventional immunosuppressive drugs and biotherapies, TCZ allowed a sustained correction of macular edema in 80% of the patients [98]. Vegas-Revenga et al. studied 25 patients with refractory macular edema, mostly JIA and Behçet’s disease. There was a reduction of macular thickness, independently of uveitis etiologies [99].

Mesquida et al. focused on the long-term efficacy of TCZ in uveitis [66]. In all 12 patients, macular edema was long-standing, on average 13 years. Sustained remission at 12 months was observed for all patients. There was macular edema relapse in all cases between 1 and 3 months after TCZ discontinuation. A re-challenge with TCZ in these patients induced recovery [66].

Most adverse events are represented by an increased risk of infections (8.5%), mostly of the gastro-intestinal tract, and increased liver enzymes (59 to 71%) with some cases of acute hepatitis and cytopenia [100].

The SATURN study has focused on sarilumab, another anti-IL6 receptor [101]. In a randomized, controlled, double-masked study, 58 patients with active (i.e., vitreous haze ≥ 4 and/or macular edema and/or vasculitis) non-anterior uveitis were included: 38 treated with sarilumab and 20 treated with placebo. The primary outcome was the improvement of vitreous haze (≥2 steps reduction) at 16 weeks. Patients in the placebo group had a longer disease course (56 versus 39 months) and had lower macular edema. At 16 weeks, 64% of the patients in the sarilumab group had reached the primary outcome, versus 35% in the placebo group (*p* = 0.04), based on investigator assessment. However, the results were not significant using fundus photographs. There was an improvement of visual acuity. Half of the patients experimented adverse events with no severe reactions: infections occurred in 25% and 26.3% of patients in the placebo group and sarilumab group, respectively. No patient had hepatic disorder or neutropenia in the placebo group, whereas these complications occurred in 7.9% of patients in the sarilumab group.

## 5. Anti-IL1 Agents

High levels of IL-1β have been identified in the aqueous humor of patients with anterior uveitis [102]. Therefore, several studies focused on IL-1β inhibition in the treatment of uveitis.

Anakinra is a recombinant monoclonal antibody that binds to the IL-1β receptor. Canakinumab is a humanized monoclonal antibody that selectively inhibits IL-1β. Anakinra and canakinumab efficacy in uveitis has been reported in Behçet’s disease. In a retrospective study of 30 patients, Emmi et al. [103] observed an efficacy of anti-IL-1 therapy in 73% of the patients and cumulative survival was 67.8% at 24 months. The median time to response to therapy was 6 weeks with anakinra and 3 weeks with canakinumab. In case of inefficacy, the switch to another anti-IL-1 therapy could improve uveitis. The same team studied 19 Behçet’s uveitis and reported that anti-IL-1 therapy improved retinal vasculitis lesions and decreased uveitis flares (from 200/100 patients/year before treatment to 48.87/100 patients/year during the 12 months of the study). However, there was no significant difference with regard to macular thickness and change of visual acuity [104]. Cantarini et al. suggested that anakinra was effective in controlling ocular inflammation but not to prevent relapse [105]. The efficacy of canakinumab in refractory JIA [106] and Blau syndrome [107] has also been reported. Most adverse events are represented by skin reactions to injections, bacterial and viral infections and neutropenia [108].

Gevokinumab, a recombinant humanized monoclonal antibody that binds to IL-1β, has been studied in a randomized controlled study of 83 non-anterior Behçet’s uveitis cases who had recently relapsed [109]. The primary outcome was the median time to relapse. Forty patients were included in the gevokinumab group and 43 patients in the placebo group. All patients received concomitant immunosuppressive drugs, either in gevokinumab and placebo groups. Macular edema was more frequent in the placebo group. All the patients were quiescent at the beginning of treatment. Gevokinumab did not significatively decrease the median time to relapse (*p* = 0.661). The rate of patients worsening visual acuity was lower in the gevokinumab group than placebo. Emergence of retinal vasculitis or macular edema was also lower in the gevokinumab group than placebo at 6 months. Most adverse events were infectious and gastrointestinal disorders. Recently, gevokinumab has been withdrawn from the market.

## 6. Anti-IL17 Agents

The only anti-IL17 agent studied in the treatment of uveitis was secukinumab, a fully human monoclonal antibody that neutralized IL-17A. Dick et al. [110] reported the results of three randomized, controlled studies: the SHIELD study (118 patients with active or quiescent non-anterior refractory Behçet’s uveitis who had experienced ≥2 relapses within 6 months), the INSURE study (31 patients with active non-anterior non-Behçet’s uveitis) and the ENDURE study (125 patients with quiescent non-anterior non-Behçet’s uveitis). In all these studies, secukinumab was administrated subcutaneously and patients had to have been previously treated with immunosuppressive drugs. The primary outcome was measured at 24 weeks in the SHIELD and ENSURE studies and at 28 weeks in the INSURE study. The dose of secukinumab ranged from 150 mg to 300 mg given either every 2 or 4 weeks. The primary end point was the reduction in rate of recurrence of uveitis in the SHIELD study, the mean change in vitreous haze in the INSURE study and the time to first recurrence of active uveitis in the ENSURE study. The three studies found no significant difference between treatment and placebo, either for ocular inflammation, relapse rate, time to relapse or improvement of visual acuity. However, secukinumab allowed a significant decrease in concomitant use of immunosuppressive drugs. Severity of uveitis might explain the absence of difference between the two groups. A few years later, Letko et al. [111] focused on the efficacy of sub-cutaneous and intra-venous (10 mg/kg every 2 weeks or 30 mg/kg every 4 weeks) secukinumab in a controlled study. The primary endpoint was the percentage of patients with treatment response (based on vitreous haze and corticosteroid sparing effect) at day 57. Thirty-three patients with non-anterior active uveitis were included. At the end of follow-up, efficacy of secukinumab was observed in 72.7% of the patients treated at a dose of 30 mg/kg IV, 61.5% of the patients treated at a dose of 10 mg/kg IV and only 33.3% of the patients treated subcutaneously. Efficacy against anterior uveitis during Behçet’s disease has been reported [112]. In a recent study, Deodhar et al. observed that the incidence rate of uveitis in patients treated with secukinumab for an ankylosing spondylo-arthritis was not increased, compared to other treatments such as anti-TNF-α agents [113].

## 7. Rituximab

Rituximab is a chimeric monoclonal antibody against CD20. Rituximab is approved in the treatment of rheumatoid arthritis and granulomatosis with polyangiitis. Ahmed et al. [114] observed an efficacy of rituximab alone, without corticosteroid, in 60% of patients (3 of 5 patients), with a mean duration of treatment of 32 months. In Lasave’s study [115], 11 patients with posterior refractory uveitis were treated with rituximab alone, without corticosteroid, during a minimal follow-up of 24 months. Rituximab was administrated at a dose of 375 mg/m2 intravenous infusion weekly for 8 consecutive weeks, and thereafter monthly for 4 consecutive months, other infusions depending on clinical evaluation. All patients had retinal vasculitis and 36% had macular edema. After 24 months of follow-up, improvement in visual acuity was observed in 38% of the patients. Relapse occurred in 24% of cases. In a retrospective study of eight patients, Miserocchi et al. [116] showed the efficacy of rituximab in JIA. At the end of follow-up (45 months), all patients had an inactive uveitis and the mean number of uveitis flares decreased from 0.7 episodes per year before rituximab to 0.2 episodes per year. In a study including Behçet’s patients with refractory macular edema or vasculitis, Davatchi et al. [117] compared two therapeutic strategies: 1/rituximab (two courses of 1000 mg at 15 days interval) plus methotrexate (15 mg/weekly) and prednisolone (0.5 mg/kg per day) or 2/cytotoxic combination therapy with pulse of cyclophosphamide (1 g/monthly), azathioprine (2–3 mg/kg per day) and prednisolone (0.5 mg/kg per day). The primary end point was the TADAI score that adds the calculation of the sum of visual acuity to TIAI (total inflammatory index of both eyes). Ten patients were included in both groups. After six months, the patients in the rituximab group reached the primary end point, but the difference was not significative between the two groups. There is a case report demonstrating the usefulness of rituximab for refractory Vogt-Koyanagi-Harada disease [118].

## 8. Abatacept

Abatacept is composed of the extracellular domain of human CTLA-4 linked to the modified Fc domain of human IgG and so blocks the CD28 costimulatory signal, necessary to T cells activation. Abatacept has been studied in JIA. In 7 patients, Zulian et al. [119] showed the efficacy of abatacept in all patients in controlling ocular inflammation and preventing relapse. Tappeiner et al. [120] studied 21 JIA patients and observed a poor control of ocular inflammation: 10% of patients at 3 months of treatment, 35% at 6 months, 57% at 9 months and only 42% at 12 months. Resolution of macular edema was observed in only 25% of cases. There was no improvement in visual acuity. All patients relapsed with abatacept when tapering off corticosteroid occurred.

## 9. Janus Associated Kinase (JAK) Inhibitors

JAK inhibitors block intracellular signal transduction downstream of different cytokine receptors, such as IL-2 and IL-6, and so appear to be an innovative and interesting option in uveitis treatment. In a mouse model of experimental dry eye disease, JAK inhibitors have shown to decrease leukocytes’ corneal infiltration and to decrease cytokine levels in the conjunctive [121]. The efficacy of tofacitinib, an anti-JAK1-JAK3 agent, has been reported to control refractory ocular inflammation [122] and macular edema [123]. Miserocchi et al. [124] have recently reported four cases of JAK inhibitor efficacy in JIA. Three patients had pan-uveitis, one had anterior uveitis and all the patients had macular edema. All patients have been previously treated with anti-TNF-α agents, three with tocilizumab and three with abatacept. One patient received tofacitinib, one received baricitinib (anti-JAK1-JAK2) as monotherapy and two received baricitinib with methotrexate. They observed an efficacy for all patients, both on ocular inflammation and macular edema.

## 10. Other Biotherapies

Some other biological agents seem to be interesting in uveitis therapeutic strategy. Targeting IL-23, a major cytokine involved in Th17 polarization, could also be an interesting therapeutic option [125]. Ustekinumab, which targets the p40 subunit that is shared by both IL-23 and IL-12, was effective in controlling uveitis associated with psoriasis [126]. Clinical trials on ustekinumab are currently ongoing: NCT02911116 and NCT01647152 in active posterior and pan-uveitis, NCT03847272 in active severe posterior and pan-uveitis with vasculitis or macular edema, and NCT02648581 in active posterior and pan-uveitis in Behçet’s uveitis. Daclizumab, a monoclonal antibody that binds to the CD25 unit of the IL-2 receptor, was studied in 17 Behçet’s uveitis patients and was not effective in preventing relapse and in tapering off immunosuppressive drugs [127]. Moreover, daclizumab was withdrawn in 2018 due to serious adverse events (encephalitis). Alemtuzumab, a monoclonal antibody anti-CD52, is responsible for T cell depletion. Mohammad et al. [128] have shown its efficacy in the sustained control of ocular inflammation in 32 Behçet’s uveitis.

The main literature results for other biological agents are reported in Table 3.

## 11. Conclusions

NIU is one of the most curable causes of blindness in developed countries. Affecting mostly young people, with an important socio-economic impact, therapeutic strategy needs to rapidly control intra-ocular inflammation, to prevent irremediable ocular damage and to allow corticosteroid sparing.

Therapeutic strategy has evolved over the last few years. Anterior NIU is mostly managed with topical treatment in adults and unilateral uveitis is mostly treated with intra-ocular injections. However, for intermediate, posterior and pan-uveitis, notably when both eyes are involved, systemic treatment is usually warranted. Biotherapies are recommended in case of inefficacy or non-tolerance of conventional immunosuppressive drugs in NINAU. Anti-TNF-α agents (ADA and IFX) are by far the most widely used.

In anterior NIU, systemic treatments are recommended in first line therapy in chronic anterior uveitis in JIA, severe sight-threatening uveitis in JIA or in case of recurrent uveitis in JIA and SA.

For NINAU, anti-TNF-α agents are used as first-line therapy in sight-threatening uveitis, with severe vasculitis or CME, particularly in Behçet’s uveitis. Data are lacking to assert superiority of ADA over IFX.

In case of treatment failure, the adherence to therapy has to be systematically questioned. Drug concentration and anti-drug antibodies to anti-TNF-α agents can identify immunization. Several strategies are possible in case of failure of one anti-TNF-α agent: switch to another anti-TNF-α agent, dose escalation, shorten interval of injection, or switch to another biotherapy (anti-IL6 agent).

Treatment duration has not been determined. Treatment discontinuation should be optimized.

Tocilizumab, an anti-IL6 agent, appears to be the best option in case of failure of anti-TNF-α agents, especially in case of CME. In view of their mechanism of action, JAK inhibitors seem to be promising in NIU.

## Figures and Tables

**Table 1:** Literature review: mains data on efficacy of anti-TNF-α agents in non-infectious uveitis.

Authors	Type of Study	Treatment	Population	Primary end Point	Num. of Patients	Results
Díaz-Llopis et al., 2012	Multicentric, open-label	ADA	Refractory uveitis to DMARDs	Efficacy of ADA on intraocular inflammation at 6 months	131	- Improvement of anterior inflammation from 1.51 to 0.25 and posterior inflammation from 1.03 to 0.14 - Improvement of BCVA (LogMAR): from mean ± SD 0.39 ± 0.44 to 0.26 ± 0.39 - Relapse rate: 38.2% - Complete resolution of macular edema: 70% - Reduction of 50% of baseline immunodepression: 85%
Dobner et al., 2013	Multicentric, retrospective	ADA	Refractory uveitis to DMARDs, mostly anterior (83%)	Efficacy	60	- Efficacy: 81.7% - Treatment discontinuation at the end of follow-up: 21.7% - Reduction of corticosteroid dose: 71.8% - Decrease of retinal thickness: 53.1%
Suhler et al., 2013	Multicentric, open-label	ADA	Refractory uveitis to DMARDs	Composite endpoint: visual acuity, inflammatory control, medication tapering and reduction of inflammatory signs at week 10	31	- Efficacy at week 10: 67.7% - Efficacy at week 52: 39%
Jaffe et al., 2016	Multicentric, randomized, placebo-controlled	ADA	Active NINAU despite corticosteroid	Time to treatment failure	217: 110 ADA and 107 placebo	- Median time to treatment failure: 24 weeks ADA group/13 weeks placebo group (*p* < 0.001) - Patients who received ADA had a significantly lower risk of treatment failure caused by vitreous haze, new active inflammatory lesions, anterior chamber cell grade, worsening of BCVA
Nguyen et al., 2016	Multicentric, randomized, placebo-controlled	ADA	Inactive cortico-dependent NINAU	Time to treatment failure	229: 115 ADA and 114 placebo	- Treatment failure rate: 39% ADA group/55% placebo group. Median time to treatment failure: >18 months ADA group/8.3 months placebo group (*p* = 0.004) - Time to treatment failure due to new active lesions, increases in anterior chamber cell grade, and increases in vitreous haze grade did not differ significantly between groups
Fabiani et al., 2017	Multicentric, retrospective	ADA	Refractory Behçet’s uveitis to DMARDs	Reduction of ocular inflammatory flares at 12 months	40	- Decrease of uveitis relapses: from 200 episodes/100 patients/year to 8.5 episodes/100 patients/year (*p* < 0.0001) - Improvement of BCVA: from 7.4 ± 2.9 to 8.5 ± 2.1 (*p* = 0.03) - Correction of CME: 69% - Improvement of retinal vasculitis: 95% - No significant difference between patients also treated with DMARDs or receiving ADA in monotherapy - No significant difference between patients treated with ADA as first line biologic therapy or second line
Mackensen et al., 2017	Multicentric, randomized, placebo-controlled	ADA	Refractory uveitis to DMARDs	Change in visual acuity (3 lines improvement) at 3 months	25: 15 ADA and 10 placebo	- Improvement of BCVA: 60% (mean increase of 0.23 logMAR) in ADA group/13% (mean increase of 0.04 logMAR) in placebo group (*p* = 0.02) - Significative improvement of ocular inflammation and CME in ADA group compared to placebo
Ramanan et al., 2017	Multicentric, randomized, placebo-controlled	ADA	Active JIA uveitis, despite MTX	Time to treatment failure	90: 60 ADA and 30 placebo	- Treatment failure rate: 27% ADA group/60% placebo group (*p* = 0.002) - Median time to treatment failure: not reached ADA group/24.1 weeks placebo group (*p* < 0.0001) - Tapering of topical glucocorticoids: 47% ADA group/ 16% placebo group (*p* < 0.0001)
Lee et al., 2018	Multicentric, retrospective	ADA	Refractory active or inactive uveitis to DMARDs	Reduction of prednisolone dose, ability to taper immunosuppressive drugs, treatment failure	22	- Reduction of dose of prednisone < 7.5 mg/day: 100% in active and inactive uveitis * Reduction of concomitant immunosuppressive drugs: 66.7% in active uveitis and 50% in inactive uveitis * Rate of treatment failure: 49.8% in active uveitis and 22.2% in inactive uveitis, mostly secondary to vitritis * Improvement of ocular inflammation: 100% in active uveitis and 50% in inactive uveitis - BCVA remained stable in both active and inactive uveitis
Quartier et al., 2018	Multicentric, randomized, placebo-controlled	ADA	Active JIA uveitis, despite MTX	Response to treatment at month 2	32: 16 ADA and 16 placebo	- Efficacy in ITT analysis: 56% ADA group/20% placebo group (*p* = 0.038) - Efficacy in PP analysis: 64% ADA group/20% placebo group (*p* = 0.015)
Suhler et al., 2018	Multicentric, open-label	ADA	Active and inactive NINAU	Quiescence at week 78	371	- 371 patients: 242 (65%) active uveitis and 129 (35%) inactive uveitis - 242 patients with active uveitis: 60% achieved quiescence at week 78, including 66% who stopped corticosteroid. Improvement of BCVA < 0.05 logMAR from 35% to 49%. Mean corticosteroid dose decreased from 13.6 mg/day to 2.6 mg/day at week 78. Mean dose of immunosuppressive drugs decreased of 26% at week 78 - 129 (35%) patients with inactive uveitis: 74% achieved quiescence at week 78, including 93% who stopped corticosteroid. BCVA remained stable. Mean corticosteroid dose remained stable. Mean dose of immunosuppressive drugs decreased of 15% at week 78
Bitossi et al., 2019	Multicentric, retrospective	ADA	Refractory uveitis to DMARDs	Control of ocular inflammation (i.e., absence of ocular flare in both eyes and reduction of the daily prednisone dose to ≤10 mg/day) at 6 months, 12 months and at the end of follow-up	105	- Ocular control: 83.7% at 6 months, 83.3% at 12 months and 94.5% at the end of follow-up (median time of 36 months). No significative difference for ocular control between patients treated only with ADA or with concomitant DMARDs - Resolution of macular edema: 77.8% - Median daily dose of prednisone: from 10 mg to 2.5 mg at 12 months (*p* < 0.001) - Rate of discontinuation: 0.15 per person-year
Tugal-Tutkun et al., 2005	Monocentric, prospective	IFX	Refractory Behçet’s uveitis to DMARDs in male patients	Remission at weeks 22 (infusion period) and at weeks 54 (observation period)	13	- Remission at week 22: 30.8% - Remission at week 54: 7.7% - Evolution of BCVA (LogMAR): from 0.56 ± 0.53 to 0.65 ± 0.72 for right eye and from 0.89 ± 0.65 to 0.68 ± 0.58 in left eye - Decrease of uveitis flare: from 2.4 ± 0.7 during the 6 months before treatment beginning to 1.0 ± 0.8 at week 22 and 1.9 ± 1.1 at week 54
Al-Rayes et al., 2008	Monocentric, open-label	IFX	Refractory Behçet’s uveitis to DMARDs	Remission: absence of uveitis attacks involving the posterior segment during the follow-up periods (3 years)	10	Remission: 30% with only 2 perfusions, 50% with a regimen of one perfusion every 8 weeks, 20% with a regimen of one perfusion every6 weeks - Improvement of anterior inflammation and visual acuity - 100% resolution of retinal vasculitis and macular edema
Suhler et al., 2009	Multicentric, open-label	IFX	Refractory NINAU to DMARDs	Composite endpoint: visual acuity, inflammatory control, medication tapering at week 10	31	- Efficacy at week 10: 77% - Efficacy at week 52: 52%
Takeuchi et al., 2014	Multicentric, retrospective	IFX	Refractory Behçet’s uveitis to DMARDs	Efficacy and relapse rate during follow-up (from 12 months to ≥ 48 months)	164	- Relapse rate for all patients: 59.1% - Relapse rate and mean time to relapse: 53.1% at 6.9 ± 4.1 months in group A (treatment duration from 12 to 23 months), 58.1 % at 7.7 ± 6.3 months in group B (treatment duration from 24 to 35 months), 54.8% in 10.4 ± 7.1 months in group C (treatment duration from 36 to 47 months), 88.2% in 10.1 ± 11.1 months in group D (treatment duration ≥ 48 months) - Decrease in ocular relapse: from 5.3 ± 3.0 to 1.0 ± 0.3 in group A (treatment duration from 12 to 23 months), 4.8 ± 4.6 to 1.4 ± 0.3 in group B (treatment duration from 24 to 35 months), 4.1 ± 2.9 to 0.9 ± 0.3 in group C (treatment duration from 36 to 47 months), 9.5 ± 5.8 to 1.6 ± 0.5 in group D (treatment duration ≥ 48 months) (*p* < 0.05 for all groups) - Improvement of BCVA: 55.8% in group A (treatment duration from 12 to 23 months), 53.8 % in group B (treatment duration from 24 to 35 months), 54.8% in group C (treatment duration from 36 to 47 months), 55.9% in group D (treatment duration ≥ 48 months)
Fabiani et al., 2017	Monocentric, retrospective	IFX	Refractory Behçet’s uveitis to DMARDs	Cumulative IFX drug retention rate during a 10-year follow- up period	40	- Cumulative IFX retention rate: 89% at 12 months, 86% at 24 months, 76% at 60 months and 47% at 120 months - 15 patients (37.5%) had discontinued IFX at the end of follow-up: 8 because of treatment failure, 4 adverse events, 2 patients for remission, 1 changed for ADA - Comparison between patients also treated with DMARDs or receiving IFX in monotherapy: no significant (*p* = 0.20) - Comparison between patients treated with IFX as first line biologic therapy or second line: significant (*p* = 0.014) - Improvement of BCVA ± SD from 7.07 ± 3.06 to 7.73 ± 3.24 at the end of follow-up (*p* = 0.047) - Median daily dose of prednisone: from 23 mg to 5 mg at the end of follow-up (*p* < 0.0001)
Maleki et al., 2017	Monocentric, retrospective	IFX	Refractory intermediate uveitis to DMARDs	Remission at 6 months	23	- Remission: 82.6%, with a mean duration of treatment to induced remission of 4 months - 34.7% of the patients stopped the treatment due to efficacy - Rate of relapse: 21.7% - Inefficacy on CME: 8.6% - Significative improvement of BCVA (*p* = 0.006) and macular thickness (*p* = 0.03) from baseline in patients who achieved remission
Ohno et al., 2019	Multicentric, retrospective	IFX	Refractory uveoretinitis to DMARDs in Behcet’s disease	Clinical response based on physician global assessment and number of ocular attacks	650	- Physician global assessment: 60.7% improved and 20.1% slightly improved - Relapse rate: 57.1% - BCVA remained stable - Median daily dose of prednisone: from 10 mg to 5 mg - Efficacy of IFX was significantly lower in patients with longer disease duration, those with comorbid diabetes mellitus, those with less severe uveitis
Martel et al., 2012	Monocentric, retrospective	ADA/IFX	Refractory uveitis to DMARDs	Sustained, corticosteroid-sparing control of inflammation at 3, 6 and 12 months	41: 12 ADA and 31 IFX	- Sustained control of inflammation with ADA: 37.5% at 3 months, 75.0% at 6 months and 57.1% at 12 months - Sustained control of inflammation plus corticosteroid-sparing success with ADA: 37.5% at 3 months, 62.5% at 6 months and 57.1% at 12 months. Median time: 151 days - Mean daily dose of prednisone with ADA: from 26.7 mg to 16.7 mg - Sustained control of inflammation with IFX: 55.6% at 3 months, 82.1% at 6 months and 69.6% at 12 months. Median time: 63 days - Sustained control of inflammation plus corticosteroid-sparing success with IFX: 33.3% at 3 months, 60.7% at 6 months and 60.9% at 12 months. Median time: 98 days - Mean daily dose of prednisone with IFX: from 22.1 mg to 12.1 mg - Overall discontinuation for ADA and IFX: 0.26 per year
Calvo-Río et al., 2014	Multicentric, open-label	ADA/IFX	Refractory Behçet’s uveitis to DMARDs	Efficacy at 12 months	124: 47 ADA and 77 IFX	- Significant reduction of both anterior inflammation and vitritis - Improvement of BCVA from 0.3 (IQR-0.1-1) to 0.8 (IQR 0.01-1) (*p* < 0.01) - Reduction of active choroiditis: 93% (*p* < 0.01) - Reduction of retinal vasculitis: 91% (*p* < 0.01) - Reduction of macular thickness from 420 microm (S.D. 119.5) to 271 (S.D. 45.6) (*p* < 0.01) - Median daily dose of prednisone: from 37.5 mg to 6.2 mg (*p* < 0.01)
Vallet et al., 2016	Multicentric, retrospective	ADA/IFX	Refractory uveitis to DMARDs	Efficacy of anti-TNF-α and the factors associated with complete response	160: 62 ADA and 98 IFX	- Rate of efficacy: 87% at 6 months, 93% at 12 months, 95% at 24 months - Incidence of complete response: 26% at 6 months, 28% at 12 months, 29% at 24 months - Event-free survival: 90% at 6 months, 70% at 12 months, 59% at 24 months - Median daily dose of prednisone: from 20 mg to 7 mg at 1 year - Factors associated with complete response to anti-TNF-α: occurrence of more than 5 relapses before initiation of anti-TNF-α treatment and Behçet’s disease - No significant difference between ADA and IFX for cumulative incidences of complete response and serious side effects
Fabiani et al., 2018	Monocentric, retrospective	ADA/IFX	Refractory NINAU to DMARDs	Efficacy of ADA and IFX at 12 months	107: 66 ADA and 41 IFX	- Decrease of 84.2% of uveitis relapses with ADA: from 168.9 episodes/100 patients/ year to 26 episodes/100 patients/year - Decrease of 66.7% of uveitis relapses with IFX: from 128.6 events/100 patients/year to 42.86 episodes/100 patients/year - Improvement of BCVA with ADA: 7.00 ± 3.62 to 7.4 ± 3.5 - Improvement of BCVA with IFX: 6.4 ± 3.4 to 6.8 ± 3.4 - Improvement of retinal vasculitis and CME with ADA and IFX without significant difference between the 2 groups (*p* = 0.51 and 0.70 respectively)
Fabiani et al., 2018	Monocentric, retrospective	ADA/IFX	Refractory NINAU to DMARDs	Long-term retention rates of ADA and IFX	108: 62 ADA and 46 IFX	- No significant difference between ADA and IFX retention rates
Atienza-Mateo et al., 2019	Multicentric, open-label	ADA/IFX	Behçet’s uveitis refractory to DMARDs	Efficacy, safety and drug retention rate	177: 74 ADA and 103 IFX	- Improvement of: * anterior chamber inflammation: 92% ADA group/78% IFX group (*p* = 0.06) * vitritis: 93% ADA group/78% IFX group (*p* = 0.04) * BCVA: mean ± SD 0.81 ± 0.26 ADA group/0.67 ± 0.34 IFX group (*p* = 0.001) * macular thickness: 250.62 ± 36.85 μm ADA group/264.89 ± 59.74 μm IFX group (*p* = 0.15) * retinal vasculitis: 95% ADA group/97% IFX group (*p* = 0.28) * drug retention rate at 1 year: 95% ADA group/85% IFX group (*p* = 0.042) * median daily dose of prednisone: from 20 mg to 5 mg at 1 year ADA group/from 30 mg to 5 mg at 1 year in the IFX group (*p* = 0.9)
Sharma et al., 2019	Multicentric, open-label	ADA/IFX	Refractory uveitis and scleritis to DMARDs	Rate of sustained remission: anterior chamber inflammation and vitreous haze scores of ≤0.5 + on two successive visits, absence of retinal vasculitis or worsening CME at 8 years	43, including 4 scleritis	- Sustained remission: 91% at a median time of 1.2 years after treatment beginning - Relapse: 51% at a median time of 2.9 years after treatment beginning - Sustained remission with ADA: 67% - Relapse with ADA: 100% - Sustained remission with IFX: 97% - Relapse with IFX: 53% - Reduction of corticosteroid dose < 7.5 mg/day: 78%
Miserocchi et al., 2013	Monocentric, retrospective	GOL	Refractory uveitis to ADA/IFX	Long-term efficacy	17	- Efficacy: 82% - Anterior relapse: 35% - Mean daily dose of prednisone: from 12.5 mg to 3.5 mg
Cordero-Coma et al., 2014	Multicentric, retrospective	GOL	Refractory uveitis to DMARDs	Efficacy at 6 months	13	- 91% of the patients previously treated with ADA or IFX - Efficacy: 92.3% - Improvement of BCVA: from 0.60 to 0.68 (*p* = 0.009) - Decrease of macular thickness: from 317 microm to 261 (*p* = 0.05)
Calvo-Río et al., 2016	Multicentric, open-label	GOL	Refractory uveitis to DMARDs in SA	Efficacy at 24 months	15	- 60% of the patients previously treated with ADA or IFX - Improvement of BCVA: from 0.62 ± 0.3 to 0.84 ± 0.3 (*p* = 0.03) - Significant improvement of intraocular inflammation (*p* = 0.04) - Improvement of macular thickness (*p* = 0.36) - Decrease in ocular relapse: from 5 [3,4,5,6] relapse/ year before treatment to 0.5 [0–3.5] relapse/year (*p* = 0.08) - Mean daily dose of prednisone: from 34.4 ± 19.4 mg to 9.2 ± 7.3 mg (*p* = 0.04)
Fabiani et al., 2017	Monocentric, retrospective	GOL	Behçet’s uveitis refractory to DMARDs and ADA/IFX	Efficacy at 12 months	5	- Efficacy: 87.5% - Improvement of retinal vasculitis: 100% - Improvement of BCVA: from 6.93 ± 4.34 to 7.32 ± 3.87
Llorenç et al., 2014	Multicentric, retrospective	CTZ	Refractory uveitis to ADA/IFX	Ocular quiescence	7	- Quiescence:71.4% - Tapering off corticosteroid: 42.8% - Improvement of visual acuity: 28.5% - Stable visual acuity: 57.1% - Improvement of macular edema (*p* = 0.021)
Rudwaleit et al., 2016	Multicentric, randomized, placebo-controlled	CTZ	Axial SA	Relapse rate	69	- Rate of uveitis relapse at week 24: 3.0 episodes/100 patient/year in CTZ and 10.3/100 patient/year in placebo - Rate of uveitis relapse at week 96: 4.9 episodes/100 patient/year
**Tosi et al., 2019**	Multicentric, retrospective	CTZ/GOL	Refractory uveitis to DMARDs	Efficacy at 12 months	21: 11 CTZ and 10 GOL	- Decrease of uveitis relapse: from 128.6 episodes/100 patients/year to 42.9 episodes/100 patients/year (*p* = 0.01)

ADA: adalimumab; DMARDs: disease modifying antirheumatic drugs, BCVA: best correct visual acuity, NINAU: uveitis non-infectious non-anterior, CME: cystoid macular edema, JIA: juvenile idiopathic arthritis, MTX: methotrexate, ITT: intention to treat, PP: per protocol, IFX: infliximab, GOL: golimumab, CTZ: certolizumab, SA: spondylo-arthritis.

**Table 2:** Summary of the various recommendations of international experts systemic therapy and anti-TNF-α use in non-infectious uveitis [2,21,37,48,51].

Uveitis Localization	Diseases	Recommendations
Anterior	JIA	- Chronic anterior uveitis requiring > 1–2 drops/eye for ≥3 months: Methotrexate - Severe active chronic anterior uveitis or sight-threatening complications: Adalimumab [51].
Anterior	SA	- Axial SA and recurrent uveitis (>3 flares/year): Adalimumab - Severe active chronic anterior uveitis or sight-threatening complications: Adalimumab [48].
NINAU	Behçet	- Always add immunosuppressive drugs (such Azathioprine) or Interferon-alpha or Infliximab to glucocorticoids - Sight-threatening uveitis: high-dose of glucocorticoids and anti-TNF-α agents. Interferon-alpha is an alternative [37].
NINAU	All	- Adalimumab for active uveitis despite corticosteroids, cortico-dependent uveitis or intolerance to corticosteroids [2,21]. - Adalimumab or infliximab for sight-threatening uveitis [2].

NINAU: non-infectious non-anterior uveitis; JIA: juvenile idiopathic arthritis; SA: spondylo-arthritis.

**Table 3:** Literature review: main data on efficacy of other biotherapies (non-anti-TNF-α agents) in non-infectious uveitis.

Authors	Type of Study	Treatment	Population	Primary End Point	Num. of Patients	Results
Calvo-Río et al., 2017	Multicentric, retrospective	TCZ 8 mg/kg IV	Refractory JIA to DMARDs and anti-TNF-α agents	Efficacy at 6 months	25	- Improvement of BCVA: from 0.56 ± 0.35 to 0.64 ± 0.32 - Improvement of vitritis: 75% - Improvement of retinal vasculitis and choroiditis: 90% - Decrease of macular thickness: from 401.7 ± 86.8 microm to 259.1 ± 39.5 microm (*p* = 0.012) - Median daily dose of prednisone: from 10 mg to 2.5 mg (*p* = 0.001)
Sepah et al., 2017	Multicentric, randomized, open-label	TCZ 8 mg/kg IV or TCZ 4 mg/kg IV	Active NINAU naive or resistant to corticosteroid or DMARDs	Incidence and severity of systemic and ocular adverse events at month 6	37: 18 (4 mg/kg) and 19 (8 mg/kg)	- 59.4% of patients were naive to prior treatment and 40.5% received corticosteroid or DMARDs - Increase of mean BCVA: mean gain of 8.22 letters ± 11.83 (ETDRS) at 6 months (*p* < 0.01) - Improvement of vitritis: 32.3% ≥ 2 steps, 77.4% ≥ 1 step - Significant decrease of macular thickness (*p* < 0.01)
Atienza-Mateo et al., 2018	Multicentric, retrospective	TCZ 8 mg/kg IV or SC	Refractory Behçet’s uveitis to DMARDs	Efficacy at 12 months	11	- 91% of the patients previously treated with ADA or IFX - Increase of mean BCVA: from 0.38 to 0.73 at the end follow-up (mean of 9.5 months) - Improvement of anterior uveitis and vitritis - Decrease of macular thickness: from 356 microm to 242 microm (*p* < 0.01) - Complete resolution of retinal vasculitis and choroiditis - Median daily dose of prednisone: from 30 mg to 0 mg (*p* = 0.005)
Eser Ozturk et al., 2018	Monocentric, retrospective	TCZ 8 mg/kg IV	Refractory Behçet’s uveitis to anti-TNF-α agents and IFN	Efficacy	5	- Improvement of anterior uveitis and vitritis for all patients - Decrease of macular thickness: 80%
Vegas-Ravenga et al., 2019	Monocentric, retrospective	TCZ 8 mg/kg IV or SC	Refractory CME to DMARDs and anti-TNF-α agents	Efficacy at 24 months	7	- Decrease of macular thickness: from 397.8 ± 232.1 microm to 231.3 ± 42.1 microm (*p* < 0.01) - Complete improvement of intra-ocular inflammation: 35.7% - Increase of mean BCVA: from 0.32 ± 0.23 to 0.59 ± 0.33 (*p* = 0.007) - Median daily prednisone dose: from 7.5 mg to 0 mg (*p* = 0.02)
Ramanan et al., 2020	Multicentric, single-arm, open-label	TCZ SC	Refractory JIA to DMARDs and anti-TNF-α agents	Treatment response at week 12: a two-step decrease, or decrease to zero, from baseline in the level of inflammation (anterior chamber cells)	21	- 29% discontinued treatment before 12 weeks, 81% discontinued treatment before 24 weeks - Treatment response at week 12: 33% (*p* = 0.11) - Resolution of macular edema: 75%
Heissigerová et al., 2019	Multicentric, randomized, placebo-controlled	SAR	Posterior uveitis refractory to corticosteroid alone or with MTX	Proportion of patients with at least a 2-step reduction in vitreous haze or with a reduction of prednisone to a dose of <10 mg/day, at week 16	58: 38 SAR and 20 placebo	- Primary outcome measured using fundus photographs: 46.1% in SAR group/30.0% in placebo group (*p* = 0.24) - Primary outcome based on investigator assessment: 64.0% in SAR group/35.0% in placebo group (*p* = 0.04) - Improvement of BCVA: mean gain of 8.9 letters (ETDRS) in SAR group/mean gain of 3.6 letters (ETDRS) in placebo group (*p* = 0.03) - Reduction of macular thickness: mean reduction of 46.8 microm in SAR group/mean increase of 2.6 microm in placebo group (*p* = 0.07)
Emmi et al., 2016	Multicentric, retrospective	ANA/CAN	Refractory Behçet’s uveitis to DMARDs	Efficacy	30: 27 ANA and 3 CAN	- Overall cumulative survival: 67.8% at 24 months - Overall cumulative survival for ANA: 31.8% at 24 months - Overall cumulative survival CAN: 40.6% at 23 months - Median time of response to therapy: 6.0 weeks for ANA and 3.0 weeks for CAN
Fabiani et al., 2017	Multicentric, retrospective	ANA/CAN	Refractory Behçet’s uveitis to DMARDs	Reduction of ocular inflammatory flares during the 12 months of treatment.	19: 13 ANA and 6 CAN	- Decrease of uveitis relapse: from 200 episodes/100 patients/year to 48.87 episodes/100 patients/year (*p* < 0.0001) - Relapse rate: 31.6% - Uveitis relapse: no difference between patients administered with ANA/CAN as first line biologic approach and those previously administered with other biologics - Improvement of retinal vasculitis: from 64.5% to 20.8% (*p* = 0.001) - BCVA and macular thickness remained stable - Mean daily dose of prednisone: from 6.11 mg to 5.8 mg (*p* = 0.02)
Tugal-Tutkun et al., 2018	Multicentric, randomized, placebo-controlled	GEV	Refractory Behçet’s uveitis to DMARDs	Reduction of ocular inflammatory flare	83: 40 GEV and 43 placebo	- All patients have quiescent disease - Relapse rate: 35% in GEV group/ 34.9% in placebo group - No significative difference in median time to first relapse - BVCA remained stable in GEV group versus aggravation in placebo group (−0.1 ± 12.2 letters in GEV group/ −3.6 ± 13.8 letters in placebo group, *p* = 0.04) - Emergence of macular edema and retinal vasculitis were less frequent in GEV group
Dick et al., 2013	Multicentric, randomized, placebo-controlled	SEC SC	- SHIELD: refractory NINAU Behçet’s uveitis to DMARDs - INSURE: active refractory NINAU to DMARDs - ENDURE: quiescent refractory NINAU to DMARDs	- SHIELD study: reduction in rate of recurrence of uveitis - INSURE study: mean change in vitreous haze - ENSURE study: time to first recurrence of active uveitis	SHIELD: 118 INSURE: 31 ENSURE: 125	- No significant difference between SEC and placebo, either for ocular inflammation, relapse rate, time to relapse or improvement of visual acuity
Letko et al., 2015	Multicentric, randomized	1/SEC SC 2/SEC 10 mg/kg IV 3/SEC 30 mg/kg IV	Refractory NINAU to DMARDs	Percentage of patients with treatment responses and percentage with complete responses (remission) at day 57	37: 1/12 2/13 3/12	- Responder rate: 33.3% SEC SC/ 61.5% SEC 10 mg/kg IV/72.7% SEC 30 mg/kg IV - Remission rate: 16.7% SEC SC/ 38.5% SEC 10 mg/kg IV/27.3% SEC 30 mg/kg IV - Median time to treatment response: 35 days SEC SC/28 days SEC 10 mg/kg IV/14 days SEC 30 mg/kg IV - Improvement of vitreous haze and decrease dose of prednisone
Davatchi et al., 2010	Multicentric, randomized	- RTX and MTX and CTC - CYC and AZA and CTC	Retinal vasculitis and edema refractive to DMARDs in Behçet’s uveitis	TADAI score that adds the calculation of the sum of visual acuity to TIAI (total inflammatory index of both eyes) after 6 months	20: 10 in both groups	- Patients in the rituximab group reached the primary end point, patients in the other did not reach the primary end point, but the difference was not significative between the two groups (*p* = 0.2) - Improvement of BCVA, macular edema and retinal vasculitis, without significant difference between the two groups
Miserocchi et al., 2016	Monocentric, retrospective	RTX	Refractory JIA to biologic agents	Efficacy	8	- Mean daily dose of prednisone: from 17.18 mg to 1.18 mg (*p* = 0.02) - Control of ocular inflammation: 100% - Decrease of uveitis relapse: from 0.7 episodes/100 patients to 0.2 episodes/100 patients
Lasave et al., 2018	Monocentric, retrospective	RTX	Refractory posterior uveitis to DMARDs	Efficacy at 24 months	11	- Control of vasculitis: 80.1% - Improvement of BVCA: 38% - Improvement of macular thickness: from 406.8 microm to 314 microm
Zulian et al., 2010	Monocentric, retrospective	ABA	Refractory JIA to anti-TNF-α agents	Efficacy at 6 months	7	- Decrease of uveitis relapse: from 3.7 episodes/100 patients to 0.7 episodes/100 patients - Relapse rate: 42.8% - Corticosteroid tapering off: 50%
Tappeiner et al., 2015	Multicentric, randomized	ABA	Refractory JIA to anti-TNF-α agents	Achievement of uveitis inactivity	21	- Uveitis inactivity: 9.5% at 3 months, 35% at 6 months, 57.1% at 9 months and 41.7% at 12 months - Relapse rate: 38% - BVCA remained stable
Miserocchi et al., 2020	Monocentric, retrospective	JAK	Refractory JIA to DMARDs and anti-TNF-α agents	Efficacy	4	- Baricitinib: 3 patients and tofacitinib: 1 patient - Resolution of ocular inflammation - Improvement of macular thickness

TCZ: tocilizumab, BCVA: best correct visual acuity, NINAU: uveitis non-infectious non-anterior, DMARDs: disease modifying antirheumatic drugs, IFN: interferon-alpha, CME: cystoid macular edema, SAR: sarilumab, MTX: methotrexate, ETDRS: Early treatment diabetic retinopathy study chart, ANA: anakinra, CAN: canakinumab, GEV: gevokinumab, SEC: secukinumab, RTX: rituximab, ABA: abatacept, JAK: JAK inhibitors.
